# Comparison of healing rate in diabetes-related foot ulcers with low frequency ultrasonic debridement versus non-surgical sharps debridement: a randomised trial protocol

**DOI:** 10.1186/1757-1146-7-1

**Published:** 2014-01-15

**Authors:** Lucia Michailidis, Cylie M Williams, Shan M Bergin, Terry P Haines

**Affiliations:** 1 Podiatry Department, Monash Health, Warrigal Road, Cheltenham, Victoria 3192, Australia; 2 Peninsula Health, Community Health, Frankston, Victoria, Australia; 3 Physiotherapy Department, Monash University, Clayton, Australia; 4 Allied Health Research Unit, Monash Health, Cheltenham, Victoria, Australia

**Keywords:** Debridement, Diabetes complications, Wound healing, Ultrasonics

## Abstract

**Background:**

Foot ulceration has been reported as the leading cause of hospital admission and amputation in individuals with diabetes. Diabetes-related foot ulcers require multidisciplinary management and best practice care, including debridement, offloading, dressings, management of infection, modified footwear and management of extrinsic factors.

Ulcer debridement is a commonly applied management approach involving removal of non-viable tissue from the ulcer bed. Different methods of debridement have been reported in the literature including autolytic debridement via moist wound healing, mechanical debridement utilising wet to dry dressings, theatre based sharps debridement, biological debridement, non-surgical sharps debridement and newer technology such as low frequency ultrasonic debridement.

**Methods:**

People with diabetes and a foot ulcer, referred to and treated by the Podiatry Department at Monash Health and who meet the inclusion criteria will be invited to participate in this randomised controlled trial. Participants will be randomly and equally allocated to either the non-surgical sharps debridement (control) or low frequency ultrasonic debridement (intervention) group (n = 322 ulcers/n = 108 participants).

Where participants have more than one ulcer, only the participant will be randomised, not the ulcer. An investigator not involved in participant recruitment or assessment will be responsible for preparing the random allocation sequence and envelopes.

Each participant will receive weekly treatment for six months including best practice podiatric management. Each ulcer will be measured on a weekly basis by calculating total area in centimetres squared. Measurement will be undertaken by a trained research assistant to ensure outcomes are blinded from the treating podiatrist. Another member of the research team will assess the final primary outcome.

**Discussion:**

The primary aim of this study is to compare healing rates for diabetes-related foot ulcers using non-surgical sharps debridement versus low frequency ultrasonic debridement over a six month period. The primary outcome measure for this study is the proportion of ulcers healed by the six month follow-up period.

Secondary outcomes will include a quality of life measure, assessment of pain and health care resource use between the two treatment modalities.

**Trial registration:**

Australian New Zealand Clinical Trial Registry: ACTRN12612000490875.

## Introduction

### Background

Diabetes is rapidly increasing in global prevalence, morbidity and mortality. In 2011, 366 million people globally were living with diabetes, a figure that is equivalent to 8.3% of the world’s adult population. It was estimated the international community would not reach this figure until 2030 [1].

In Australia the prevalence of type 2 diabetes has increased over the past two decades and continues to rise. Approximately 7% of the Australian population is thought to have type 2 diabetes and it is estimated that 15% of people with diabetes will develop a foot ulcer during their lifetime [2]. The consequences of having diabetes in Australia are significant with over 500,000 hospital admissions and 12,000 deaths attributed to the condition in 2004 alone [1].

The pathophysiology of foot ulceration is complex and usually multi-factorial. Peripheral sensory neuropathy, foot deformity and external trauma, when occurring concurrently, have been identified as being the three most common factors that predispose to diabetes-related foot ulcers (DRFU) [3]. Peripheral arterial disease has also been shown to lead to the development of ischaemic and neuro-ischaemic DRFU [2]. Regardless of the true aetiology, the same complications can arise with all DRFU, including soft tissue infection, osteomyelitis, tissue necrosis and failure of ulcer healing, all of which may require hospital admission and potentially result in amputation [4].

Diabetes has been acknowledged to be the most common cause of non-traumatic lower-limb amputation in Australia [5]. Furthermore, acute complications affecting foot ulceration have been reported as the leading cause of diabetes-related hospital admissions and amputation [4]. For the years 2004–2005 the Australian Institute of Health and Welfare reported that DRFU resulted in 9900 acute hospital admissions [4]. In the same period 3400 diabetes-related lower limb amputations were also reported [2].

More recently it has also been suggested that diabetes-related lower limb amputations have increased by 30% between the years 1998 – 2005 [6]. The estimated acute care cost of a single lower extremity amputation in Australia could be as much as $26,700 [4]. This figure does not include costs for rehabilitation, purchase of orthotics/prosthetics or time lost from work. Recent economic evaluations of the cost of a lower limb amputation for a single person found that Australia sits in third place behind France where such a procedure is estimated to cost $46,064 for a single diabetes-related lower extremity amputation and in Germany the same is estimated at a cost of $31,809 [2]. The cost of amputation secondary to diabetes complications in the United States of America is said to range from $20,000 - $60,000 per patient and similarly does not include the personal, social, or economic aspects of the patient’s life [7].

None of the costs noted above consider the direct financial burden on patients with a DRFU. The ongoing costs of ulcer management in the community have not been investigated in the literature to date, however clinicians, patients and their families feel the impact of these costs every day. It has been reported however, that in one study investigated the quality of life of patients with DRFU 50% of patients were no longer in work because of their ulcer. Although treatment was free the costs associated with travelling to hospital appointments and buying additional footwear [8] placed an additional financial strain on patients.

Given the complications associated with DRFU and the time these ulcers can take to heal it is not surprising that patients report a greatly reduced quality of life [9]. It has been found that all quality of life domains can be adversely impacted primarily because of a reduction in mobility and the consequent need to adapt activities of daily living [8]. Additionally it is thought that the presence of a foot ulcer imposes restrictions on patient participation and enjoyment of their usual hobbies mainly because of mobility difficulties and the requirements for treatment [9]. This has been shown to have a negative psychological effect with an increase in patients with depression and a lower satisfaction with their personal lives [9]. Reviewing and improving ulcer management interventions that have the potential to result in more effective and faster healing could have the added benefit of improving the quality of life of patients with a DRFU.

Debridement has been identified as a leading treatment for management of DRFU [2]. Debridement has been defined as the removal of devitalised, contaminated or foreign material from within or adjacent to the ulcer until surrounding healthy tissue is exposed [10]. It serves several functions including reduced pressure on the ulcer base, more thorough inspection to determine true ulcer depth and size, facilitation of drainage and creation of an acute ulcer environment [6].

Existing approaches to ulcer debridement can be performed directly by a clinician including theatre-based sharps debridement (TBSD) also known as surgical excision and non-surgical sharps debridement (NSSD) or scalpel debridement in a clinical setting. There are also various topical products that act as debriding agents. These have included wet-dry dressings that act as mechanical debriding agents, dressings that encourage moist wound healing and autolytic debridement, biological debridement through use of sterile larvae and also the use of chemical enzymes [10-13].

Theatre-based sharps debridement has been utilised for removal of deep necrotic tissue, gangrene and deep infection [14] but has not been routinely used as part of standard care. Non-surgical sharps debridement is required more regularly to remove non-viable necrotic tissue from the ulcer surface and is recommended as part of standard ulcer care [13]. The need for and appropriate method of ulcer debridement should be determined based on the clinical presentation [12] and potentially the clinical skillset and equipment available [13].

Sonoca 185™ (SÖering) was introduced in Australia recently as an alternative method for ulcer debridement. The technology works by delivering low frequency ultrasound, or sound waves, through a constant flow of saline. Ultrasound results when electrical energy is converted to sound waves at frequencies above the range of human hearing (20 kHz) with Sonoca 185™ functioning at 25 kHz [15]. These sound waves can then be transmitted to tissue, via a liquid medium, through a treatment applicator. It is the non-thermal effects of ultrasound that have been shown to cause two phenomena at the ulcer surface; acoustic streaming [15-17] (a steady mechanical force delivered in a fluid medium i.e. sterile saline) and cavitation [15-17] (formation of gas bubbles in the fluid creating micro-shockwaves). The combined effects of acoustic streaming and cavitation are thought to alter cell membrane activity and increase the activity of each cell [16]. Subsequently this is thought to have three clinical effects: debridement, a bactericidal effect and an ulcer healing stimulator effect [17-19].

The biological effects indicated through in vitro and animal studies could contribute to ulcer healing [20]. These effects include stimulation of cellular activity and protein synthesis, the activation of inflammatory cells and the production of chemical mediators that activate fibroblasts and may lead to ulcer healing [15,19,20]. Additionally the mechanical forces produced by the ultrasound energy at the cellular and molecular levels may promote ulcer healing by fostering cell division, angiogenesis, the release of growth factors [20] and stimulating collagen synthesis [15,19]. In vitro data has also found that low frequency ultrasonic debridement (LFUD) is effective in reducing microbe count for methicillin-resistant staphylococcus aureus, vancomycin resistant enterococci, pseudomonas and other commonly occurring bacteria [17,18].

When comparing LFUD with TBSD significant clinical advantages have been noted in terms of efficacy and safety for debriding ulcers without deep infection or necrosis. Successful TBSD is reliant upon the skill of the surgeon and their ability to distinguish between tissue types. Procedural risks of TBSD have included pain, bleeding [21], damage to underlying structures with a resultant loss of function [13,22], post-surgical infection and the use and associated risks of general anaesthesia [13].

Comparisons have been made with the use of LFUD and TBSD in DRFU in a randomised controlled trial, which found a mean healing rate that was 2.5 times faster using LFUD compared to TBSD over a two week treatment period. Limitations of this study include the very short follow-up of only two weeks and the small sample size (N = 59) [23].

A randomised double-blind controlled trial has compared low-frequency low-intensity ultrasonic debridement to a sham treatment (saline mist without ultrasound) in patients with recalcitrant DRFU. Ennis et al. found that after 12 weeks of treatment 40.7% of patients who underwent LFUD had healed compared to only 14.3% in the sham treatment group. Whilst this is promising data the overall numbers of participants were small (N = 55) [24].

A recent meta-analysis investigating the use of non-contact low-frequency high-intensity ultrasonic debridement, reported significant improvement compared to NSSD at three and five months, but no difference at six months. There were only two studies suitable for the meta-analysis, one focused on DRFU (N = 40) and the other venous leg ulcers (N = 76). Again the overall numbers were small [16].

Another meta-analysis concluded that non-contact LFUD is an efficacious treatment for chronic wounds of varying aetiologies [20]. Despite the quality of the initial evidence being of low quality suggests that LFUD does demonstrate short-term clinical benefits when used as an adjunctive therapy. Recommendations from both meta-analyses were the same; there is no evidence that compares LFUD with standard ulcer management. Additionally, there is a need for further research using larger randomised clinical trials of longer period of time.

Given the evidence available it could be expected that LFUD might be a lower-cost treatment when compared to TBSD in terms of the cost associated with the actual treatment itself and potential savings from healing ulcers faster.

Non-surgical sharps debridement has been considered the leading comparator to TBSD for several reasons; the technique is simple and requires the use of basic instruments by a trained professional; it is time efficient and can be performed in clinic or at the bed-side; does not require the resources of an operating theatre and has a lower overall cost.

Evidence on the most appropriate method, frequency and extent of DRFU debridement is limited and insufficient to draw any conclusions. The National Evidence-Based Guidelines for the Prevention, Identification and Management of Foot Complications in Diabetes recommends that NSSD should be considered first and should occur repeatedly and as often as required to remove all non-viable tissue [2]. This recommendation is based on expert opinion in the absence of evidence pertaining to DRFU debridement.

A recent Cochrane Review [10] on debridement of diabetic foot ulcers notes that while ulcer debridement is recommended as an effective intervention to assist healing, no guidelines identify a specific method of debridement. The methods of debridement reviewed included surgical debridement, topical hydrogels and larval therapy [10]. Neither NSSD nor LFUD were investigated in the Cochrane Review.

The method of choice for ulcer debridement remains inconclusive. Evidence suggests that each ulcer needs to be individually assessed in terms of type, size, position, appearance, patient pain and tolerance, cost effectiveness and available expertise and equipment to determine the most suitable method of debridement [25].

The decision to utilise NSSD as the active control group in this study was based on the expert opinion in clinical guidelines and the low cost and easy accessibility of the treatment for clinicians. The limited data around LFUD leaves a gap in the evidence that warrants further investigation. The limited data available on LFUD with NSSD as standard practice makes this debridement modality a choice comparator.

It is hypothesised that use of LFUD in the treatment of DRFU would improve healing rates when compared with NSSD. There will be four aims within this study. The primary aim is to determine if there is a difference in healing rates for DRFU, using NSSD compared to LFUD. Secondary aims include assessing for differences in pain during and post-treatment, determining if there is a difference between the quality of life of participants who have an ulcer undergoing either method of debridement and if there is a difference in overall costs between NSSD and LFUD.

This clinical trial will provide important information in the field of ulcer management; provide a better understanding of the efficacy of NSSD and the newer technology of LFUD. It will also provide health services with a better understanding of the financial impacts of both treatments. This protocol has been designed and reported to ensure it corresponds to the 33 items of the Standard Protocol Items: Recommendations for Interventional Trials (SPIRIT) checklist [26].

## Methods

### Study design

**Figure 1:**
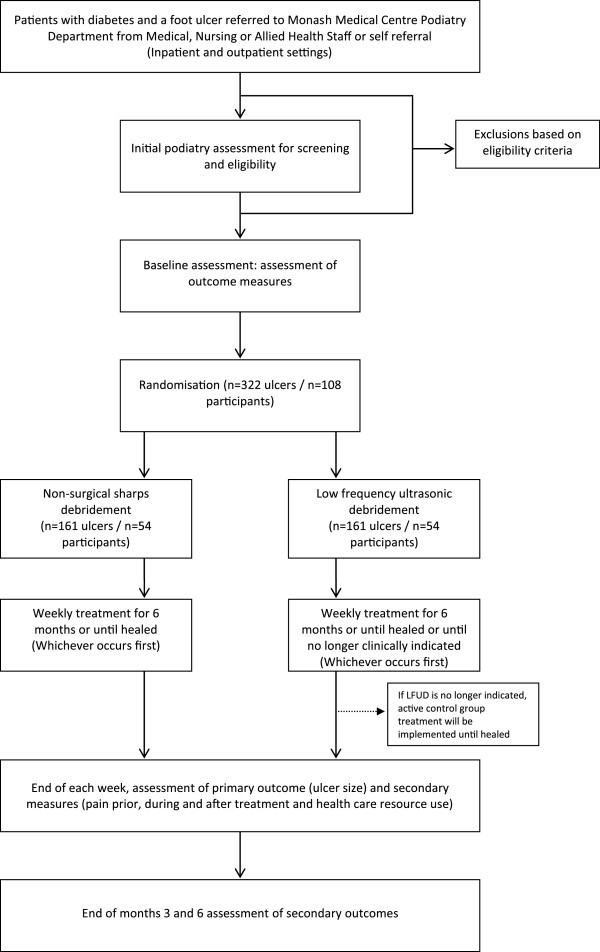
Consort flow chart for the study.

This is a randomised controlled trial comparing NSSD (active control group) and LFUD (treatment group) in DRFU with a six month follow-up period. A consort flow chart for the design of this study is presented in Figure 1.

### Ethical consideration

Ethical approval for this study has been obtained by the Monash Health Human Research Ethics Committee HREC 12101B.

### Participants and setting

Patients with diabetes and a foot ulcer/s, who are referred to and treated by the Podiatry Department at Monash Health, will be invited to participate in this study. Patients may be inpatients and receiving podiatry care on the ward or outpatients referred by the patient’s primary medical care team.

This study is a single centre trial. The average length of stay for an acute hospital admission in Australia is 6 days [27]. Participants may be recruited during their hospital admission but it is anticipated they will receive treatment primarily in the outpatient setting. Inpatients, however, can receive either treatment if they meet inclusion criteria for this study as both study interventions can be undertaken by the bedside as well as in an outpatient clinical setting.

A standard initial podiatric assessment will occur at baseline including a neurovascular assessment, medical and surgical history, medications history, diabetes management and control history including glycated haemoglobin (Hba1c), footwear assessment, ulcer aetiology, ulcer duration and previous management. If the participant meets the inclusion criteria (Table 1) as determined by the treating podiatrist, the patient will be informed about the research project and written consent will be obtained to participate in the study.

**Table 1:** Participant inclusion and exclusion criteria

**Inclusion criteria**	**Exclusion criteria**
***General:***	***General:***
≥ 30 years of age	Patients taking immunosuppressive medications
Able to provide informed consent	Known allergy to ulcer dressing products
Ulcers present for greater than 1 month	Pre-existing ulcer pain preventing either type of debridement
Ulcers ≥ 1 cm^2^	
***Vascular:***	***Vascular:***
Palpable pedal pulses OR biphasic or triphasic pedal pulses on doppler OR toe pressure ≥ 45 mmHg	Non-palpable pedal pulses OR monophasic pedal pulses on Doppler OR toe pressure ≤ 45 mmHg
***Ulcer classification:***	***Ulcer Classification:***
Infected ulcers being appropriately managed	Dry gangrenous ulcer
***Those meeting The University of Texas Wound classification criteria *** [28] ***:***	Fungating ulcers
*A1, A2, A3 (wounds of varying depth without infection or ischaemia)*	Malignant ulcers
*B1, B2, B3 (wounds of varying depth with infection only)*	***Those meeting the University of Texas wound classification criteria *** [28] ***:***
	*A0, B0, C0, D0 (pre or post-ulcerative lesion with complete epithelialisation, with or without infection and ischaemia)*
	*C1, C2, C3 (wounds of varying depth with ischaemia only)*
	*D1, D2, D3 (wound of varying depth with infection and ischaemia)*

Ulcers must be chronic, or greater than 1 month in duration to be included in the study. This is to capture the most accurate data around DRFU, which have been shown in the literature to take longer than 4 weeks to heal [29]. Should a patient have an ulcer infection at the time of recruitment, or develop an infection during the trial they will receive appropriate antibiotic therapy and will be able to continue in the trial. If appropriate infection management is not commenced, irrespective of the reasons, the patient will not be able to continue in the trial.

### Interventions

The two interventions are the two different methods of ulcer debridement, LFUD and NSSD. The techniques for both treatments are as described in Table 2.

**Table 2:** Standard step-by-step technique for LFUD and NSSD

**LFUD**	**NSSD**
1) Constantly move the handpiece to prevent ultrasound burning tissue	1) Start debriding at the distal most aspect of the ulcer
2) Start debriding at the distal most aspect of the ulcer	2) Moving scalpel proximally with each motion
3) Moving the handpiece left to right and from the distal to proximal aspect of the ulcer	3) Once the distal to proximal ulcer has been debrided then debride from left side to right side
4) Once the entire ulcer surface has been debrided re-commence the same technique from the distal most aspect of the ulcer	4) Continue until as much necrotic tissue has been removed as possible
	5) Any peri-wound tissue that requires removal (i.e. callus, maceration) will occur using a scalpel.
5) Continue until as much necrotic tissue has be removed as possible	
6) Any peri-wound tissue that requires removal (i.e. callus, maceration) will occur using a scalpel. The wound base will not be debrided with the scalpel.	

### Outcome measures

#### Primary outcome measure

The primary outcome measure for this study is the proportion of ulcers healed over the six month follow-up period. An ulcer is defined as healed in the presence of intact skin, i.e. functional epithelial tissue [30], a total surface area of 0 cm^2^ and restoration of functional and anatomic continuity [31]. Ulcer healing status will be determined by assessing the total ulcer area.

Ulcer surface area will be assessed using photographs taken with a digital camera using a standard technique (Table 3). A one centimetre by one centimetre, transparent grid will be utilised over the printed photograph and the total area calculated. Total surface area measurements will be performed following each weekly treatment. A research assistant blinded to the treatment allocation will collect the data for the primary outcome measure. This is to ensure the treating podiatrist is blinded to the primary outcome during subsequent treatments.

**Table 3:** Standard step-by-step technique for ulcer measurement

**Ulcer measurement**	
1)	Ulcers that have tunnels or undermining will be marked on the skin with a black marker
2)	A white towel will be place under the foot to remove distracting background elements
3)	A disposable ruler will be labelled with participant number, wound number, participant initials and the date
4)	Position the disposable ruler alongside the ulcer and secure with paper tape
5)	Use macro camera setting with flash on, iso set to 200
6)	Take photograph at a distance of 20 cm from the wound
7)	Ulcer measurements will be conducted from print out using the photograph (all photos will be printed as standard A4 size)

The research assistant has been trained by the treating podiatrist and given written instructions on how to use the transparent grid to calculate total ulcer area. To determine reliability fifteen ulcers have been photographed and both the research assistant and treating podiatrist followed the same technique to calculate ulcer area. Inter-rater measurement reliability between the treating podiatrist and research assistant was found to have an ICC of 0.91.

The ulcer depth will be measured by the treating podiatrist following each treatment, as depth cannot be accurately assessed using a photograph. A disposable measurement probe will be used to assess ulcer depth, undermining, sinus or tracking.

A review of available literature around ulcer measurement is scarce and of low evidence. The measurement technique being used in this study, tracing and subsequent counting of centimetre squares, has a high inter-rater and intra-rater reliability when compared to other forms of ulcer measurement [32,33].

A standard technique will be used for each method of debridement and ulcer measurement ensuring consistency (Tables 2 and 3).

Ulcers being treated in the intervention group will be reviewed after six weeks of treatment. If LFUD is no longer clinically indicated then treatment will be ceased and the ulcers will then receive the control treatment (NSSD). This change is to reflect the pragmatic nature of the treatment and NSSD is considered standard ulcer care. Clinical indications for ceasing LFUD treatment include pain, ulcer size and depth, clinical presentation and no ulcer improvement.

#### Secondary outcome measures

Secondary outcome measures will include assessing ulcer pain, quality of life and economic evaluation.

Ulcer pain will be measured weekly using a 100 mm Visual Analogue Scale (VAS). Pain will be assessed prior to, during and following each treatment. The far left end of the scale (0 mm) will be labelled as no pain and the far right end of the scale (100 mm) will be labelled as worst pain imaginable. The VAS has been widely used and has been shown to be a valid and reliable pain assessment tool [34].

A health-related quality of life tool will be used to gain perspective from each participant. This will be undertaken at the initial treatment, at three months and again at six months. If an ulcer heals prior to the end of the six month study period the tool will be applied at that point. The Eq 5D-5 L [35] assessment tool analyses five health-related quality of life domains including mobility, self-care, usual activities, pain/discomfort and anxiety/depression. This tool has been widely used and has been validated for use in patient groups with diabetes [36].

All data for the secondary outcome measures will be collected by the treating podiatrist. No blinding will occur for this data.

Each outcome measure and their time points of collection are summarised in Table 4.

**Table 4:** Outcome measures and timeframes

***Data collection***	***Measurement tool***	***Data collected method***	***Timeframe***
*Measurement of total ulcer area*	*Centimetres squared;*	*Research assistant*	*Weekly: Post-treatment until healed or at 6 months*
	*Tracing from photographs and counting squares*		
*Measurement of ulcer depth*	*Centimetres;*	*Treating podiatrist*	*Weekly: post-treatment until healed or at 6 months*
	*Using sterile probe*		
*Ulcer pain*	*Visual analogue pain scale 100 mm*	*Treating podiatrist*	*Weekly: Pre-treatment, during treatment, post-treatment until healed or 6 months*
*Quality of life*	*EQ-5D-5 L tool*	*Participant questionnaire*	*Initial treatment, at 3 months, at 6 months*
***Direct health costs***			
*Consumable costs for treatments*	*In dollars for each treatment*	*Treating podiatrist*	*Weekly, per participant until healed or at 6 months*
*Medicare Benefit Scheme (MBS)*	*MBS Care database, in dollars*	*Extraction from MBS database*	*End of project for each participant from initial to final treatment*
*Pharmaceutical Benefit Scheme (PBS)*	*PBS Care database, in dollars*	*Extraction from PBS database*	*End of project for each participant from initial to final treatment*
*Inpatient data*	*Monash Health:*	*Hospitalisation costs*	*Monash Health:*
	*Admission duration, reason for admission, imaging and interventions, obtained from the patient record and from the Victorian Admitted Episodes Database*		*End of project*
			*External organisation: End of project*
	*External organisation: Admission duration, reason for admission, costs of any surgery for diabetes-related foot ulcers will be estimated using WEISS funding*		
*Hospital based services (outpatient data)*	*Hours – time spent*	*Treating podiatrist*	*Weekly per participant until healed or at 6 months*
*Medical imaging and pathology for outpatients*	*Dollars – hospital based costs*	*Treating podiatrist*	*Monthly per participant until healed or at 6 months*
*Community based services*	*Number and cost of appointments*	*Participant interview*	*Monthly until healed or at 6 months*
*Private health appointments*	*Number and cost of appointments, eligibility for private health subsidies*	*Participant interview*	*Monthly until healed or at 6 months*
*Royal District Nursing Service for ulcer management*	*Frequency and cost of service*	*Participant interview*	*Monthly until healed or at 6 months*
*Ongoing ulcer care products*	*Valued using market prices*	*Participant interview*	*Monthly until healed or at 6 months*
*Parking costs for appointments*	*Dollars*	*Participant interview*	*Monthly until healed or at 6 months*
*Transportation costs to travel to appointments*	*Estimated through Australian Tax Office car rate cents per km*	*Participant interview*	*Monthly until healed or at 6 months*
***Productivity costs***			
*Time taken from work for participant and/or any family member*	*Salary and hours taken from work*	*Participant/family interview*	*Monthly until healed or at 6 months*

### Sample size

The sample size calculation for this study was based upon the primary outcome comparison between groups of the proportion of ulcers completely healed by the six month follow-up. Previous research indicates that nearly 25% of ulcers treated with NSSD healed within six months [29], while another previous study found that 41% of ulcers treated with LFUD healed within three months [24]. There is no six month data available for the LFUD approach. A sample size of 147 ulcers per group is required to achieve 80% power using a two-tailed alpha of 0.05 to detect an absolute difference in the proportion of ulcers healed of 0.16 (control = 0.25, intervention = 0.41). To account for the intra-cluster correlation of multiple ulcers being nested within a single participant we adjust this for a design effect (1 + (n-1)*ICC) using n = 3 ulcers per participant and ICC estimate of 0.05; thus we require 161 ulcers per group. With an average of 3 ulcers per participant we require 54 participants per group.

### Randomisation

Randomisation will be undertaken using a permuted-block randomisation approach. Randomisation blocks of two, four or eight participants will be generated and randomly selected and the resultant allocation order will be entered into opaque, sealed envelopes. An investigator not involved in recruitment or assessment (CW) will be responsible for preparing the random allocation sequence and envelopes. The treatment conditions will be provided as per the random allocation sequence following completion of the initial assessment.

Once eligibility has been confirmed, a verbal explanation of the project will be provided and the treating podiatrist will obtain written consent. All participants who consent will have baseline assessments conducted prior to randomisation, as outlined above. All ulcers (where there is more than one per participant) will be numbered and documented according to anatomical location prior to randomisation. Only the treatment condition will be randomised, not each individual ulcer. Where there is more than one ulcer, all will be treated with the same method as per the randomisation process and included in the study. Following randomisation the initial treatment and measurements will commence as outlined in Tables 2 and 3. All participants will receive treatment and have their ulcers photographed and measured on a weekly basis, as is standard podiatry practice at Monash Health. Both groups will receive best practice ulcer management including appropriate ulcer dressings, pressure off-loading and footwear provision as required.

Identifiable outcome data will be stored within the participant’s health record. De-identifiable data will be stored within a password-protected Excel spread sheet within a secure hospital data management system as per requirement of the Human Research Ethics Committee (HREC) for Monash Health. The primary investigator (LM) will be responsible for data entry and a co-investigator (SB) will randomly audit information to monitor data accuracy.

The trial will be managed by the research team and led by the primary investigator (LM). The protocol has undergone external review from the Lions John Cockayne Research Fellowship committee and the research team will give quarterly progress reports. Annual reports will also be required (including adverse events) to the HREC of Monash Health. The research team will meet on a monthly basis to address clinical and data monitoring concerns.

### Statistical analysis

The proportion of ulcers that are completely healed by the six month follow-up will be compared between groups using a logistic regression analysis approach with clustering of ulcer within participant. A member of the research team (TH) who will be blinded to the allocation of the participants will assess this.

The rate of change in ulcer size (surface area, using the post-debridement photo) will be compared between groups using a linear mixed model analysis approach where repeated assessments will be nested within ulcer, and ulcers will be nested within participants. The groups will be treated as a fixed factor while assessments, ulcer and participants will be treated as random factors. All analyses will be adjusted for whether the wound was infected at baseline, as infection has been demonstrated to delay healing [37] and HbA1c levels at baseline as poor glycaemic control has been demonstrated to delay healing [38].

A pre-planned interim analysis will be undertaken after 70% of the planned sample size has been recruited. This analysis will use all data available to that point in time and examine the safety and efficacy outcomes from the trial. A data analyst who is blinded to group allocation will be provided with the dataset and mock group codes. The outcome of this analysis will be forwarded to the remaining project investigators who will decide whether there is sufficient evidence to reject the null hypothesis for the primary outcome. The assumptions underlying the sample size calculation (e.g. ICC value) will also be examined at this point and revisions to the sample size will be made if indicated.

### Economic analysis

#### Cost effectiveness analysis

Direct and indirect health care costs will be collected at regular intervals, as explained in Table 3.

The formula for assessing cost effectiveness analysis will be:

$$\frac{{\mathrm{Cost}}_{\mathrm{LFUD}}–{\mathrm{Cost}}_{\mathrm{NSSD}}}{{\mathrm{Effect}}_{\mathrm{LFUD}}–{\mathrm{Effect}}_{\mathrm{NSSD}}}=\mathrm{Incremental}\;\mathrm{cost}\;\mathrm{per}\;\mathrm{additional}\;\mathrm{ulcer}\;\mathrm{healed}$$

#### Cost utility analysis

A health related quality of life assessment obtained from the EQ-5D-5 L tool will be converted to utility scores as explained in Table 4. The economic evaluation will examine the cost per quality adjusted life year (QALY) gained per patient provided with each intervention. QALY measurements will use the EQ-5D-5 L utility-based cost-effectiveness analysis. The formula to calculate QALYs gained from the intervention will be:

$$\frac{{\mathrm{Cost}}_{\mathrm{LFUD}}–{\mathrm{Cost}}_{\mathrm{NSSD}}}{{\mathrm{QALY}}_{\mathrm{LFUD}}–{\mathrm{QALY}}_{\mathrm{NSSD}}}=\mathrm{Incremental}\;\mathrm{cost}\;\mathrm{per}\;\mathrm{QALY}\;\mathrm{gained}$$

## Discussion

Diabetes-related foot ulceration is a significant medical and social problem. Consensus among wound specialists supports the importance of ulcer debridement to encourage ulcer healing. Despite this, there is a paucity of evidence comparing different debridement techniques. Whilst there is evidence available around the efficacy of LFUD it has been limited. Furthermore, there is no randomised controlled trial looking at the healing rates of DRFU that undergo NSSD compared to LFUD.

This clinical trial will provide important information in the field of ulcer management and provide a better understanding of the efficacy of using NSSD treatment. It will also provide health services with a better understanding of the financial impacts of both treatments.

Adverse events will be measured and recorded during the study. The adverse events for both treatment groups may include incidents such as sharps injuries to the participant or treating podiatrist, development of ulcer infection, hospital admission due to ulcer deterioration, excess pain and bleeding from debridement at the ulcer surface.

A limitation of this study is the non-consideration given to nutritional status. Patient nutritional status has potential to impact on ulcer healing, however outside of a controlled inpatient environment it is difficult to enforce a strict food regime. All patients will be encouraged to adhere to a suitable diet, however this will not be controlled as part of this study.

A second limitation is that while a thorough assessment of pain will be undertaken, this measure will only focus on the individual ulcer pain before, during and after debridement with either modality. Where participants have more than one ulcer in close proximity to another ulcer the pain assessment may become difficult to distinguish for each ulcer.

## Abbreviations

NSSD: Non-surgical sharps debridement; LFUD: Low frequency ultrasonic debridement; TBSD: Theatre-based sharps debridement; DRFU: Diabetes-related foot ulceration.

## Competing interests

The authors declare no competing interests.

## Authors’ contributions

All the authors contributed to study design and methodology. LM and CW obtained funding for the study. All authors contributed to the study protocol. LM is the chief investigator and drafted the paper. SB, TH, and CW provided editorial assistance. All authors have read and approved of the final paper.
